# Cancer stigma and cancer screening attendance: a population based survey in England

**DOI:** 10.1186/s12885-019-5787-x

**Published:** 2019-06-11

**Authors:** Charlotte Vrinten, Ailish Gallagher, Jo Waller, Laura A. V. Marlow

**Affiliations:** Cancer Communication and Screening Group, Department of Behavioural Science and Health, UCL, Gower Street, London, WC1E 6BT UK

**Keywords:** Cancer, Stigma, Screening, Early detection, Prevention, Bowel, Breast, Cervical

## Abstract

**Background:**

Cancer-related stigma attracts considerable research interest, but few studies have examined stigmatisation in the healthy population. Qualitative studies suggest that stigma can discourage people from attending cancer screening. We aimed to quantify the prevalence and socio-demographic patterning of cancer stigma in the general population and to explore its association with cancer screening attendance.

**Methods:**

In 2016, 1916 adults aged 18–70 years took part in home-based interviews in England. Measures assessed demographic characteristics, self-reported screening uptake for cervical (*n* = 681), breast (*n* = 326) and colorectal cancer (*n* = 371), and cancer stigma. Cancer stigma was measured with the validated Cancer Stigma Scale which assesses six subdomains (*Severity*, *Personal Responsibility, Awkwardness, Avoidance, Policy Opposition,* and *Financial Discrimination*), from which a mean score was calculated*.* Logistic regression analyses examined the association between cancer stigma and having been screened as recommended versus not.

**Results:**

Levels of cancer stigma were low, but varied across the six subdomains. Items regarding the severity of a cancer diagnosis attracted the highest levels of agreement (30–51%), followed by statements about the acceptability of making financial decisions on the basis of a cancer diagnosis such as allowing banks to refuse a mortgage (16–31%) and policy opposition statements such as not having a responsibility to provide the best possible care for cancer patients (10–17%). A similar proportion anticipated feeling awkward around someone with cancer (10–17%). Only 8–11% agreed with personal responsibility statements, such as that a person with cancer is to blame for their condition, while 4–5% of adults anticipated avoiding someone with cancer. Stigma was significantly higher in men (*p* < .05) and in those from ethnic minority backgrounds (*p* < .001). Higher cancer stigma was associated with not being screened as recommended for all three screening programmes (cervical: adjusted OR 1.59, 95% CI 1.15–2.20; breast: adjusted OR = 1.97, 95% CI 1.17–3.32; colorectal: adjusted OR = 1.59, 95% CI 1.06–2.38).

**Conclusions:**

Cancer stigma is generally low, but some aspects of stigma are more prevalent than others. Stigma is more prevalent in certain population subgroups and is negatively associated with cancer screening uptake. These benchmark findings may help track and reduce cancer stigma over time.

**Electronic supplementary material:**

The online version of this article (10.1186/s12885-019-5787-x) contains supplementary material, which is available to authorized users.

## Background

Despite recent improvements in treatment and survival, cancer is still seen as a stigmatised disease. Cancer can remind people of their own mortality and present uncomfortable connotations of death and suffering, leading to feelings of awkwardness and fear [1]. Indeed, recent surveys show that a quarter of people in the UK believe that a cancer diagnosis is a death sentence [2, 3], and half of the population believes that cancer treatment is worse than cancer itself [3].

Health-related stigma is defined as “a social process or related personal experience characterised by exclusion, rejection, blame or devaluation that results from experience or reasonable anticipation of an adverse social judgment about a person or group identified with a particular health problem” [4]. Although this definition emphasises the felt experience of stigma by the stigmatised, stigma can be studied in two ways: from the perspective of the stigmatisers (also referred to as “public” or “enacted” stigma) and from the perspective of the stigmatised (i.e. “felt stigma” or “self-stigma”) [1, 5, 6]. Most research into cancer stigma thus far has focused on the latter, the stigma felt by cancer patients, with the majority of studies examining stigma in lung cancer patients [7–10]. These studies show that lung cancer patients who are current or former smokers tend to feel particularly stigmatised due to the perception that their illness is self-inflicted and they are therefore to blame for it [11].

Cancer stigma may not just affect cancer patients, but public stigma of cancer may also negatively impact public health efforts to reduce the burden of cancer in the wider society. With 1 in 2 people born after 1960 in the UK expected to develop some form of the disease during their lifetime [12], cancer is high on the public health agenda. However, exploratory work suggests that expectations and fear of being stigmatised might discourage some people from engaging in cancer prevention or early detection because it may result in discovering you belong to a stigmatised group [7, 13]. Various studies have found that negative beliefs about cancer are indeed associated with lower screening uptake [14, 15], lower rates of self-examination for skin cancer [16], and higher healthcare avoidance for fear of having the illness [17]. The hypothesis that stigma may deter help seeking for illness is consistent with a number of studies in other fields that have reported how anticipated stigma can impede help seeking for mental health [18–21], sexual health/HIV [22, 23], cirrhosis [24], and dementia [25].

Few studies have systematically explored public cancer stigma. A study in more than five thousand cervical screening-eligible women in Ireland found that mean anticipated stigma scores for 8 items about HPV infection were just below the midpoint (2.3 on a scale from 1 to 4), but were higher for those with lower levels of education [26]. However, the scale consisted of various stigma-related items, such as personal responsibility for disease, avoidance, and disgust, and scores for individual items were not reported, making it impossible to assess whether there is variation in agreement across the items. An online US survey that did report scores for individual components of stigma found that agreement with statements about personal responsibility for cancer were generally low (mean score 2.4 out of 7), agreement with statements of societal responsibility for cancer were mid-range (3.7/7), and that willingness to engage in various prosocial cancer-related behaviours, such as donating money to a cancer charity, was relatively high (4.7/7) [27]. However, the sample may not have been representative of the general US population (for example, more than half reported college-degree level education, compared with a 30% national average [28]), and results may not be generalisable to other populations outside the US.

The Cancer Stigma Scale (CASS) is a validated scale that was developed to measure the multiple dimensions of cancer stigma in a non-patient population [29]. It has six distinct domains: three factors relating to people’s perceptions of cancer and three factors encompassing social aspects and anticipated behaviour towards cancer patients. Previous research has used the CASS to look at stigma in the general population in the UK, but the data were from an online panel, limiting the generalisability of the findings [30]. This study aimed to i) assess prevalence and socio-demographic patterning of cancer stigma in a population-representative sample of English adults, and ii) examine the association between cancer stigma and self-reported uptake of breast, cervical, and colorectal cancer screening in those eligible for screening.

## Methods

### Participants

Data were collected as part of the cross-sectional Attitudes, Behaviour and Cancer UK Survey (ABACUS) in 2016, from which other studies have been published (e.g. [31]). Adults aged 18–70 years (*N* = 2048) were eligible to participate. We chose the lower age limit to ensure maturity for informed consent to participate in the survey, while the upper age limit represents the age when people cease being invited to cancer screening. Participants were recruited as part of the Kantar TNS omnibus survey, during several waves in April and May 2016. Kantar TNS is a market research agency that uses random location sampling to recruit participants. The sampling points are defined based on the 2011 census small area statistics and postcode address file. Quotas are set by Kantar TNS at each location for gender or housewife, children in the household, and working status. Kantar TNS also supplies weights to ensure population-representativeness. Kantar TNS collects data for their omnibus survey using face-to-face home-based computer assisted interviews. Ethical approval was granted by the University College London (UCL) ethics committee (Project ID Number: 5771/002). All participants gave verbal consent at the start of the omnibus interview, which was recorded on the TNS database as part of the interview.

### Measures

#### Cancer stigma

Cancer stigma was measured using items from the Cancer Stigma Scale (CASS) [29], a validated scale with 25 items assessing six subdomains*: Awkwardness, Severity, Avoidance, Personal Responsibility, Policy Opposition* and *Financial Discrimination.* Due to space restrictions we selected 18 items (3 per subscale), based on an online pilot survey (*n* = 392) with quotas for age, gender and educational status. In the pilot, we examined the item-total correlations for the items in each subscale to assess possible redundancy, and retained items with the highest correlation for the main survey. Responses were recorded on a 6-point Likert scale from ‘disagree strongly’ to ‘agree strongly’ and reversed scored for 4 items, so that all scores were from 1 to 6 with higher scores indicating more stigma (see Table 2 for items that were reverse scored). For the descriptive statistics, we dichotomised the responses into “agree” (agree slightly, agree moderately, and agree strongly) and “disagree” (disagree slightly, disagree moderately, disagree strongly) and present the percentage of people agreeing to each item. In addition, we calculated mean scores for each of the subscales (excluding those who had more than one missing item on that subscale), as well as a total mean score for all 18 items of the CASS (with those missing more than 6 items excluded from analyses). The presentation of means for each subscale allows for the comparison of our results with the original CASS development paper [29]. Note that the scale as used in the current study measures stigma of cancer in general (i.e. as a group of diseases), and is not specific to any one type of cancer.

In the present sample, internal reliability of the whole scale (0.77, and 0.88 in the pilot) and four of the subscales was somewhat lower than in the online pilot survey, but still adequate: avoidance (0.71, 0.90 in the pilot), personal responsibility (0.74, 0.90 in the pilot), policy opposition (0.71, also 0.71 in the pilot) and severity (0.71, 0.75 in the pilot). Despite good internal reliability for these scales in the online pilot survey (financial discrimination 0.82 and awkwardness 0.81), Cronbach’s alpha was lower for financial discrimination (0.60) and awkwardness (0.57) in the current sample.

#### Cancer screening uptake

Cancer screening is offered via the National Health Service (NHS) in England with eligibility based on age and gender: breast: women aged 50–70; cervical: women aged 25–64; colorectal (using the faecal occult blood test): men and women aged 60–70. Past attendance at cancer screening was self-reported and only asked to those eligible for that screening programme at the time of interview. Responses were dichotomised for analysis as “screened as recommended” (i.e. those who had been every time they were invited for that particular screening programme) and “not screened as recommended” (i.e. those who had not been every time they were invited and those who had never been despite being eligible). Those who refused to answer this question were coded as missing.

#### Socio-demographics

Sociodemographic variables included age, gender, ethnicity, and social grade. Ethnicity was assessed using the 2011 census question [32] recoded into “White” (any white background) and “Black, Asian, and Minority Ethnic (BAME)”. Social grade was recoded into four categories using the National Readership Survey (NRS) social grades, which are based on the occupation of the household’s chief income earner “AB” (higher and intermediate managerial, administrative and professional), “C1” (supervisory, clerical and junior managerial administrative and professional), “C2” (skilled manual workers), and “DE” (semi-skilled and unskilled manual workers, and state pensioners, casual and lowest workers, unemployed with state benefits only) [33].

### Analyses

We excluded those with missing data on the sociodemographic variables and those with a previous diagnosis of cancer or who refused to say whether they had previously been diagnosed with cancer, because they were not asked the questions about cancer stigma to avoid unnecessary distress. For the analyses on screening uptake, we also excluded those with missing data on the uptake variables. The sample characteristics are reported using weighted and unweighted data. The percentage agreement with each cancer stigma item, as well as mean scores per sub scale, were reported using weighted data to help accurately estimate prevalence. We then looked at the sociodemographic predictors of the total cancer stigma score using linear regression analyses, both in univariable and multivariable analyses adjusting for other sociodemographic variables (age, gender, ethnicity and social grade). For those analyses, we used the unweighted data because weights do not tend to alter the associations and using the unweighted data is recommended because it is simpler and reduces the risk of overfitting [34]. Finally, we examined the association between total cancer stigma score and uptake of breast, cervical and colorectal cancer screening using logistic regression analyses with the unweighted data, both unadjusted and adjusted for all socio-demographic variables. As a sensitivity analysis, we also examined the association between total cancer stigma score and screening uptake, dichotomising the uptake variable as “having never been screened” vs “having been screened at least once”. All statistical analyses were carried out in SPSS 24, using a significance level of *p* < .05 to indicate statistical significance, except for the sociodemographic associations where we use *p* < .001 to allow for multiple testing.

## Results

### Sample characteristics

**Fig. 1:**
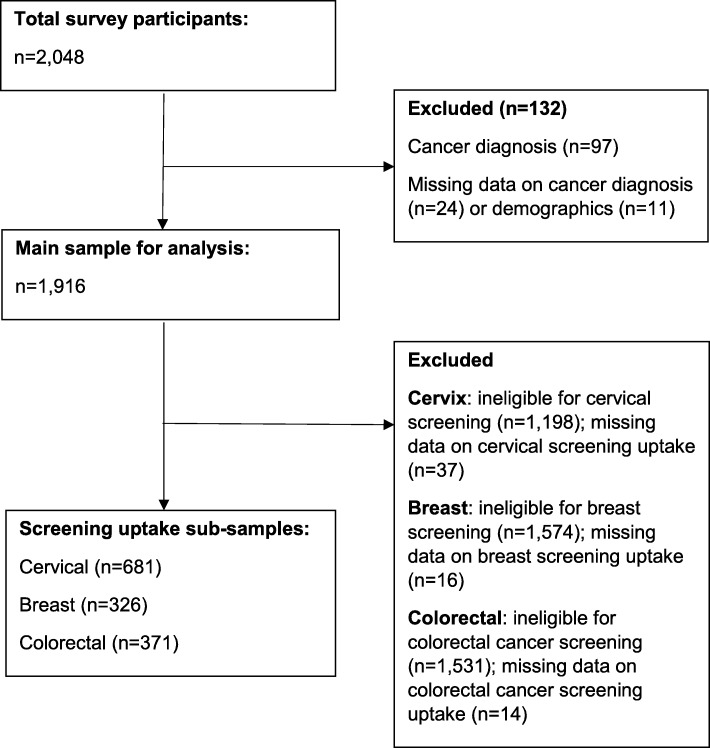
Flow chart of participant inclusion and exclusion

Overall 2048 adults completed the survey. Figure 1 shows the number of participants excluded because of a cancer diagnosis or missing data, which resulted in a sample of 1916 for analyses. Sample characteristics are displayed in Table 1. Mean age of the weighted sample was 42.8 (*SD* = 15.3), about half (51%) were female, and the majority of the sample were from a White ethnic background (84%). About a quarter were from the highest social grade (AB; 27%), half were from the middle grades (C1: 28% and C2: 22%) and 23% belonged to the lowest grades (DE).

**Table 1:** Unweighted and weighted sample characteristics, and unweighted sociodemographic associations of total CASS score

	Unweighted^a^	Weighted^a^	Total Cancer Stigma Univariable			Total Cancer Stigma Multivariable^c^	
	*N* = 1916	*N* = 1918	N = 1823			N = 1823	
	N (%)^b^	N (%)^b^	M (SD) ^d^	B (SE) ^d^	β^d^	B (SE) ^d^	β^d^
Total sample			2.10 (0.57)				
Age in years (M, SD)	43.0 (15.9)	42.8 (15.3)					
18–34	703 (36.7)	681 (35.5)	2.11 (0.59)				
35–54	648 (33.8)	731 (38.1)	2.11 (0.57)	−.003 (.032)	−.003	.006 (.032)	.005
55–70	565 (29.5)	506 (26.4)	2.08 (0.55)	−.035 (.033)	−.028	−.007 (.034)	−.005
Gender							
Female	1025 (53.5)	971 (50.6)	2.06 (0.54)				
Male	891 (46.5)	946 (49.4)	2.15 (0.60)	.088 (.027)	.077*	.088 (.027)	.077*
Ethnicity							
White (Any)	1610 (84.0)	1614 (84.1)	2.07 (0.55)				
BAME	306 (16.0)	304 (15.9)	2.29 (0.64)	.225 (.037)	.142*	.219 (.037)	.138*
Social grade							
AB (high)	329 (17.2)	511 (26.7)	2.05 (0.56)				
C1	551 (28.8)	537 (28.0)	2.08 (0.55)	.023 (.040)	.018	.014 (.040)	.011
C2	420 (21.9)	428 (22.3)	2.10 (0.55)	.046 (.043)	.034	.046 (.042)	.033
DE (low)	616 (32.2)	442 (23.0)	2.15 (0.61)	.100 (.040)	.082	.098 (.039)	.080

*BAME* Black, Asian, and Minority Ethnic

*p < .001

^a^ The weighted and unweighted sample sizes are unequal because weights were calculated based on the whole sample (*N* = 2048) before applying exclusions for the current analyses

^b^Unless otherwise stated

^c^ Adjusted for age, gender, ethnicity and social grade

^d^ *M* mean, *SD* standard deviation, *B* unstandardized regression coefficient, *SE* standard error, *β* standardised regression coefficient

### Cancer stigma

Completion rates, agreement with each cancer stigma item and means for each sub scale using the weighted sample are reported in Table 2. Completion rates for the individual cancer stigma items were high at around 95% of the sample. Levels of cancer stigma were low but varied across the six sub scales. Items regarding the severity of a cancer diagnosis attracted the highest levels of agreement (30–51%; M = 3.08, SD = 1.07). This was followed by statements about the acceptability of making financial decisions on the basis of a cancer diagnosis, such as allowing banks to refuse a mortgage, to which 16–31% agreed (M = 2.31, SD = 1.03). Policy opposition statements were endorsed by 10–17% (M = 2.10, SD = 1.05), while a similar proportion (10–17%) would feel awkward around someone with cancer (M = 1.85, SD = 0.95). Only 8–11% agreed with personal responsibility statements, such as that a person with cancer is to blame for their condition (M = 1.81, SD = 0.90), while 4–5% of adults anticipated avoiding someone with cancer (M = 1.40, SD = 0.72).

**Table 2:** Completion rates and agreement with each of the cancer stigma items for the weighted sample (N = 1918)^a^

	Completion rate N (%)	Agree N (%)	M (SD)^b^
Severity			3.08 (1.07)
Having cancer usually ruins a person’s career	1826 (95.2)	825 (45.2)	
Getting cancer means having to mentally prepare oneself for death	1832 (95.5)	937 (51.1)	
Cancer usually ruins close personal relationships	1830 (95.4)	547 (29.9)	
Financial discrimination			2.31 (1.03)
It is acceptable for banks to refuse to make loans to people with cancer	1829 (95.4)	316 (17.3)	
Banks should be allowed to refuse mortgage applications for cancer-related reasons	1826 (95.2)	285 (15.6)	
It is acceptable for insurance companies to reconsider a policy if someone has cancer	1827 (95.3)	567 (31.0)	
Policy opposition			2.10 (1.05)
More government funding should be spent on the care and treatment of those with cancer (R)	1824 (95.1)	1552 (85.1)	
The needs of people with cancer should be given top priority (R)	1816 (94.7)	1500 (82.6)	
We have a responsibility to provide the best possible care for people with cancer (R)	1832 (95.5)	1641 (89.6)	
Awkwardness			1.85 (0.95)
I would feel comfortable around someone with cancer (R)	1829 (95.4)	1526 (83.4)	
I would find it difficult being around someone with cancer	1824 (95.1)	174 (9.5)	
I would find it hard to talk to someone with cancer	1826 (95.2)	204 (11.2)	
Personal responsibility			1.81 (0.90)
A person with cancer is liable for their condition	1819 (94.8)	193 (10.6)	
If a person has cancer it’s probably their fault	1828 (95.3)	139 (7.6)	
A person with cancer is to blame for their condition	1824 (95.1)	160 (8.8)	
Avoidance			1.40 (0.72)
I would feel angered by someone with cancer	1826 (95.2)	83 (4.5)	
I would try to avoid a person with cancer	1831 (95.5)	79 (4.3)	
If a colleague had cancer I would try to avoid them	1826 (95.2)	67 (3.7)	

^a^(R) denotes items that were reverse coded for calculation of the mean scores

^b^*M* mean, *SD* standard deviation

### Sociodemographic associations with cancer stigma

We examined the associations between sociodemographic variables and cancer stigma using the total cancer stigma score for the unweighted sample. For these analyses, we excluded a further 93 participants (4.9%) who had missing data on more than 6 items of the CASS. Total cancer stigma scores in the remaining sample (*N* = 1823) were low (mean CASS score = 2.10 (out of a possible 6), SD = 0.57; Table 1).

Linear regression analysis showed that higher stigma scores were associated with being male (M = 2.15, SD 0.60 vs M = 2.06, SD 0.54; β = .077, *p* < .001) and being from an ethnic minority background (M = 2.29, SD 0.64 vs M = 2.07, SD 0.55; β = .138, p < .001, for the multivariable analysis), both in univariable and multivariable analyses adjusting for other sociodemographic variables. Stigma was not significantly associated with age or social grade.

### Association between cancer stigma and cancer screening participation

**Fig. 2:**
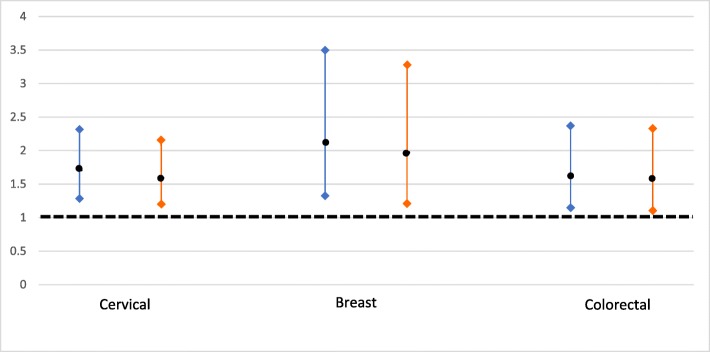
Unadjusted (left) and adjusted (right) odds ratios and 95% confidence intervals for not being screened as recommended (versus being screened as recommended) by total CASS score (continuous)

The association between cancer stigma and self-reported screening uptake was examined in the subgroups of those with complete data on the sociodemographic, cancer stigma score, and cancer screening uptake variables who were eligible for cervical (*n* = 681), breast (*n* = 326), and colorectal (*n* = 371) cancer screening (see Fig. 2). Overall, 26% of eligible participants were not screened as recommended for cervical screening (*n* = 175), 18% for breast screening (*n* = 57), and 33% for colorectal cancer screening (*n* = 124).

In unadjusted analyses, higher total cancer stigma was significantly associated with increased odds of not being screened as recommended for all three screening programmes: cervical OR = 1.71 95% CI: 1.25–2.34, *p* < .001; breast OR = 2.12, 95% CI: 1.28–3.54, *p* < .01; and colorectal cancer screening OR = 1.63, 95% CI: 1.10–2.42, *p* < .05. These associations remained significant after adjusting for all sociodemographic variables: cervical OR = 1.59 95% CI: 1.15–2.20, p < .01; breast OR = 1.97, 95% CI: 1.17–3.32, p < .05; and colorectal cancer screening OR = 1.59, 95% CI: 1.06–2.38, p < .05 (see Fig. 2). The results of the sensitivity analyses, examining the association between cancer stigma and screening uptake of those who had been screened at least once versus those who had never been screened, were very similar and are reported in the Additional file 1.

## Discussion

This is the first population-representative study to show that cancer stigma in English adults is generally low, but is higher in men and those from ethnic minority backgrounds and is negatively associated with cancer screening. In addition, stigma varied by subdomain, with lowest endorsement of statements regarding avoidance, awkwardness, and personal responsibility, but higher endorsement of statements about policy opposition, acceptability of financial discrimination and severity of a cancer diagnosis.

This was the first study to use the CASS in a population-representative sample in the UK and thus serves as a benchmark for cancer stigma in the general population in England. Compared with the CASS development paper (which used an online panel sample of a similar age range and gender distribution in England in 2010, but had a slightly higher proportion of participants from ethnic minority backgrounds) [29], the mean scores for each of the subscales in our sample were very similar, except for the awkwardness and avoidance scores, which were somewhat lower in our sample. It is difficult to interpret these differences; they may be due to sampling effects, mode effects (face-to-face interview versus online survey), or they may indicate that feelings of awkwardness and the desire to avoid cancer patients are decreasing over time. It should be noted that levels of avoidance as assessed in the current study are very low, with only 4–5% of the general population anticipating avoiding someone with cancer, although feelings of awkwardness were slightly higher and endorsed by 10–17%.

The demographic pattern of cancer stigma was also similar to that found in the CASS development paper [29], with higher levels of cancer stigma in men than women, and among those from ethnic minority backgrounds compared with those from White ethnic backgrounds. To our knowledge, no other studies have explicitly examined gender differences in public cancer stigma, although two studies in university student samples suggest that women are less likely than men to distance themselves from patients with cancer or refuse to help them [35, 36]. The finding that cancer stigma is higher in men is also consistent with a study about mental health stigma among US legislators that found that men were overrepresented among legislators with high levels of stigma (84% male versus 75% of all legislators included in the study) [37].

There is some previous research on cancer stigma in ethnic minority communities, although mainly in qualitative studies, where participants often describe cancer as a stigmatised disease, a ‘taboo’ or as not openly discussed within their communities [38–41]. A narrative review identified cancer stigma among ethnic minority communities as a barrier to accessing cancer genetic services [42, 43], and Black cancer patients expressed a greater need for post-treatment information on how to deal with stigma than White patients [44]; findings that are indicative of greater cancer stigma in ethnic minority communities. There may be a need for culturally sensitive interventions designed especially to address stigma in ethnic minorities.

Unlike previous findings [26], we did not find an association between socioeconomic status and cancer stigma, but comparisons between these studies are difficult due to differences in samples, stigma measures, and operationalisation of socioeconomic status. There is well-documented socioeconomic variation in cancer risk behaviours and mortality [45, 46], which may affect perceptions of cancer stigma. Future studies should further examine the association between cancer stigma and socioeconomic status, in particular for the specific subscales of cancer stigma, such as personal responsibility and severity attributions, and their association with cancer prevention and early detection.

We also found that higher stigma scores were associated with not being screened as recommended for all three types of cancer screening. This is consistent with previous research findings. For example, qualitative studies in the US and Australia identified cancer stigma as a barrier to lung cancer screening and help-seeking for possible lung cancer symptoms [47, 48]. Lung cancer stigma was also associated with delayed help-seeking in a quantitative US study among recently-diagnosed lung cancer patients, even after controlling for other variables associated with delayed presentation such as ethnicity, smoking status, and medical distrust [13]. Unlike the US, lung cancer screening has not yet been implemented in the UK, and future studies may wish to explore the effect of cancer stigma on lung screening uptake, especially because previous research suggests that public stigma of lung cancer is greater than for other common types of cancer, such as breast, colorectal, and skin cancer [30].

About one in ten people endorsed statements about personal responsibility for cancer. It is estimated that about 40% of cancers are due to lifestyle choices [49], and public health campaigns are increasingly raising awareness of this link between modifiable risk factors and cancer to further the cancer prevention effort. However, an unintended consequence of this may be that cancer could increasingly be seen as being self-inflicted. This kind of “victim blaming” may already be happening for lung cancer patients because of the well-known association with smoking [8, 50], but may become apparent for other cancers as well once other modifiable cancer risk factors, such as obesity, poor diet, and alcohol consumption, become better known to the general public. Ironically, increased stigma due to better awareness may negatively impact engagement with cancer prevention and so it is imperative for public health campaigners to find the “sweet spot” for maximising cancer risk factor awareness while minimising stigma [50]. Future studies could help by monitoring the evolution of the different dimensions of stigma over time, in particular the personal responsibility and policy opposition dimensions.

Our study had several limitations. First, we had to reduce the number of items in the CASS due to space restrictions in the survey, but this may have lowered the internal validity of some of the subscales. Second, the ABACUS survey is a population-representative survey but no response rate is recorded by the market research agency. This means that the levels of cancer stigma reported here, and the percentage endorsement of the individual items, can be generalised to the population of English adults aged 18–70. However, the ABACUS survey was not powered to examine the association between cancer stigma and cancer screening uptake, and the strength of the associations found here may be limited by the smaller sample sizes. In addition, due to the small samples eligible for each of the cancer screening programmes, we could not examine the association between the 6 separate dimensions of cancer stigma and screening uptake. Furthermore, the items in the CASS are about cancer in general. Previous research has shown that some cancer types elicit more stigma than others [30]. Different types of cancer also have different aetiologies, and the degree to which the public is aware of this varies, so future studies should consider trying to unpick associations between knowledge, stigma and behaviour for specific cancer types [1]. However, we expect that the associations found between cancer stigma and cancer screening uptake, which were statistically significant in both unadjusted and adjusted analyses, would be even stronger if cancer type-specific measures of stigma were used. Another limitation of the current study is that the internal reliability of some of the shortened sub scales of the CASS was quite low. Despite our best efforts to create an internally reliable shorter version of the CASS by conducting some pilot work, we recommend that future studies use the full set of 25 CASS items. Alternatively, more work could be done to develop a shorter version of the CASS that has better internal reliability, although any attempts to shorten the CASS would necessarily be restricted by the number of sub scales that need to be accurately measured. Finally, this was a cross-sectional study so no inferences about causality between cancer stigma and cancer screening attendance can be made. Future studies should prospectively include screening-eligible samples that are large enough to examine differences in screening uptake for each of the 6 dimensions of cancer stigma, and may wish to adapt the CASS to make it cancer-specific.

## Conclusions

Cancer stigma in England is generally low, but still exists, with some aspects more prevalent than others. Stigma is greatest among particular population sub-groups, including those from ethnic minority backgrounds, and is negatively associated with cancer screening behaviours. Our benchmark findings may help track and reduce cancer stigma in England over time.

## Additional file

Additional file 1: **Table S1.** Unadjusted and adjusted odds ratios (OR), 95% confidence intervals (95% CI), and significance values for having never been screened (vs. having been screened at least once) by total CASS (cancer stigma) score for cervical (*N* = 681), breast (*N* = 326) and colorectal cancer screening (*N* = 371). (DOCX 14 kb)

## Data Availability

Data available from the first author upon reasonable request.

## Abbreviations

ABACUS: Attitudes and Behaviour about Cancer UK Survey
BAME: Black And Minority Ethnic
CASS: Cancer Stigma Scale
NHS: National Health Service
NRS: National Readership Survey
OR: Odds Ratio

## References

[1] Else-Quest N, Jackson T, Corrigan PW (2014). Chapter 8: Cancer stigma. The stigma of disease and disability: understanding causes and overcoming injustices.

[2] Agustina E, Dodd RH, Waller J, Vrinten C (2018). Understanding middle-aged and older adults' first associations with the word "cancer": a mixed methods study in England. Psychooncology..

[3] Quaife SL, Winstanley K, Robb KA, Simon AE, Ramirez AJ, Forbes LJ (2015). Socioeconomic inequalities in attitudes towards cancer: an international cancer benchmarking partnership study. Eur J Cancer Prev.

[4] Weiss MG, Ramakrishna J (2006). Stigma interventions and research for international health. Lancet..

[5] Jones N, Corrigan PW, Corrigan PW (2014). Chapter 1: Understanding stigma. The stigma of disease and disability: understanding causes and overcoming injustices.

[6] Goffman E (1963). Stigma: notes on the management of spoiled identity.

[7] Knapp Sarah, Marziliano Allison, Moyer Anne (2014). Identity threat and stigma in cancer patients. Health Psychology Open.

[8] Lebel S, Devins GM (2008). Stigma in cancer patients whose behavior may have contributed to their disease. Future Oncol.

[9] Marlow LA, Waller J, Wardle J (2010). Variation in blame attributions across different cancer types. Cancer Epidemiol Biomark Prev.

[10] Else-Quest NM, LoConte NK, Schiller JH, Hyde JS (2009). Perceived stigma, self-blame, and adjustment among lung, breast and prostate cancer patients. Psychol Health.

[11] Chambers SK, Dunn J, Occhipinti S, Hughes S, Baade P, Sinclair S (2012). A systematic review of the impact of stigma and nihilism on lung cancer outcomes. BMC Cancer.

[12] Ahmad AS, Ormiston-Smith N, Sasieni PD (2015). Trends in the lifetime risk of developing cancer in Great Britain: comparison of risk for those born from 1930 to 1960. Br J Cancer.

[13] Carter-Harris L, Hermann CP, Schreiber J, Weaver MT, Rawl SM (2014). Lung cancer stigma predicts timing of medical help-seeking behavior. Oncol Nurs Forum.

[14] Miles A, Rainbow S, von Wagner C (2011). Cancer fatalism and poor self-rated health mediate the association between socioeconomic status and uptake of colorectal cancer screening in England. Cancer Epidemiol Biomark Prev.

[15] Ndukwe EG, Williams KP, Sheppard V (2013). Knowledge and perspectives of breast and cervical cancer screening among female African immigrants in the Washington D.C. metropolitan area. J Cancer Educ.

[16] Michielutte R, Dignan MB, Sharp PC, Boxley J, Wells HB (1996). Skin cancer prevention and early detection practices in a sample of rural women. Prev Med.

[17] Moser RP, Arndt J, Han PK, Waters EA, Amsellem M, Hesse BW (2014). Perceptions of cancer as a death sentence: prevalence and consequences. J Health Psychol.

[18] Corrigan P (2004). How stigma interferes with mental health care. Am Psychol.

[19] Villatoro AP, DuPont-Reyes MJ, Phelan JC, Painter K, Link BG (2018). Parental recognition of preadolescent mental health problems: does stigma matter?. Soc Sci Med.

[20] Clement S, Schauman O, Graham T, Maggioni F, Evans-Lacko S, Bezborodovs N (2015). What is the impact of mental health-related stigma on help-seeking? A systematic review of quantitative and qualitative studies. Psychol Med.

[21] Gary FA (2005). Stigma: barrier to mental health care among ethnic minorities. Issues Ment Health Nurs.

[22] Marcell AV, Morgan AR, Sanders R, Lunardi N, Pilgrim NA, Jennings JM (2017). The socioecology of sexual and reproductive health care use among young urban minority males. J Adolesc Health.

[23] Rade DA, Crawford G, Lobo R, Gray C, Brown G (2018). Sexual health help-seeking behavior among migrants from sub-Saharan Africa and South East Asia living in high income countries: a systematic review. Int J Environ Res Public Health.

[24] Vaughn-Sandler V, Sherman C, Aronsohn A, Volk ML (2014). Consequences of perceived stigma among patients with cirrhosis. Dig Dis Sci.

[25] Herrmann LK, Welter E, Leverenz J, Lerner AJ, Udelson N, Kanetsky C (2018). A systematic review of dementia-related stigma research: can we move the stigma dial?. Am J Geriatr Psychiatry.

[26] O'Connor M, O'Leary E, Waller J, Gallagher P, Martin CM, O’Leary JJ (2018). Socio-economic variations in anticipated adverse reactions to testing HPV positive: implications for the introduction of primary HPV-based cervical screening. Prev Med.

[27] Myrick JG (2017). Public perceptions of celebrity cancer deaths: how identification and emotions shape cancer stigma and behavioral intentions. Health Commun.

[28] United States Census Bureau. Quick Facts. Available from: https://www.census.gov/quickfacts/fact/table/US/PST045217. Accessed 6 June 2019.

[29] Marlow LA, Wardle J (2014). Development of a scale to assess cancer stigma in the non-patient population. BMC Cancer.

[30] Marlow LA, Waller J, Wardle J (2015). Does lung cancer attract greater stigma than other cancer types?. Lung Cancer.

[31] Murphy PJ, Marlow LAV, Waller J, Vrinten C (2018). What is it about a cancer diagnosis that would worry people? A population-based survey of adults in England. BMC Cancer.

[32] Office for National Statistics. 2011 Census questionnaire for England. Available from: https://www.ons.gov.uk/census/2011census/howourcensusworks/howwetookthe2011census/howwecollectedtheinformation/questionnairesdeliverycompletionandreturn. Accessed 6 June 2019.

[33] National Readership Survey. Social Grade. Available from http://www.nrs.co.uk/nrs-print/lifestyle-and-classification-data/social-grade/. Accessed 6 June 2019.

[34] Korn EL, Graubard BI (1991). Epidemiologic studies utilizing surveys: accounting for the sampling design. Am J Public Health.

[35] Schulte A (2002). Consensus versus disagreement in disease-related stigma: a comparison of reactions to AIDS and cancer patients. Sociol Perspect.

[36] Mosher CE, Danoff-Burg S (2007). Death anxiety and cancer-related stigma: a terror management analysis. Death Studies.

[37] Purtle J, Le-Scherban F, Wang X, Shattuck PT, Proctor EK, Brownson RC (2018). Audience segmentation to disseminate behavioral health evidence to legislators: an empirical clustering analysis. Implement Sci.

[38] Randhawa G, Owens A (2004). The meanings of cancer and perceptions of cancer services among south Asians in Luton, UK. Br J Cancer.

[39] Thomas VN, Saleem T, Abraham R (2005). Barriers to effective uptake of cancer screening among black and minority ethnic groups. Int J Palliat Nurs.

[40] Bedi M, Devins GM (2016). Cultural considerations for south Asian women with breast cancer. J Cancer Surviv.

[41] Jones CE, Maben J, Lucas G, Davies EA, Jack RH, Ream E (2015). Barriers to early diagnosis of symptomatic breast cancer: a qualitative study of black African, black Caribbean and white British women living in the UK. BMJ Open.

[42] Allford A, Qureshi N, Barwell J, Lewis C, Kai J (2014). What hinders minority ethnic access to cancer genetics services and what may help?. Eur J Human Genet.

[43] Hann KEJ, Freeman M, Fraser L, Waller J, Sanderson SC, Rahman B (2017). Awareness, knowledge, perceptions, and attitudes towards genetic testing for cancer risk among ethnic minority groups: a systematic review. BMC Public Health.

[44] Asare M, Peppone LJ, Roscoe JA, Kleckner IR, Mustian KM, Heckler CE (2018). Racial differences in information needs during and after cancer treatment: a nationwide, longitudinal survey by the University of Rochester cancer center national cancer institute community oncology research program. J Cancer Educ.

[45] Lian M, Schootman M, Doubeni CA, Park Y, Major JM, Stone RA (2011). Geographic variation in colorectal cancer survival and the role of small-area socioeconomic deprivation: a multilevel survival analysis of the NIH-AARP diet and health study cohort. Am J Epidemiol.

[46] Sauer AG, Siegel RL, Jemal A, Fedewa SA (2017). Updated review of prevalence of major risk factors and use of screening tests for cancer in the United States. Cancer Epidemiol Biomark Prev.

[47] Carter-Harris L, Ceppa DP, Hanna N, Rawl SM (2017). Lung cancer screening: what do long-term smokers know and believe?. Health Expect.

[48] Crane M, Scott N, O'Hara BJ, Aranda S, Lafontaine M, Stacey I (2016). Knowledge of the signs and symptoms and risk factors of lung cancer in Australia: mixed methods study. BMC Public Health.

[49] Parkin DM, Boyd L, Walker LC (2011). 16. The fraction of cancer attributable to lifestyle and environmental factors in the UK in 2010. Br J Cancer.

[50] Riley KE, Ulrich MR, Hamann HA, Ostroff JS (2017). Decreasing smoking but increasing stigma? Anti-tobacco campaigns, public health, and cancer care. AMA J Ethics.

