# Supplementary material for: Cancer stigma and cancer screening attendance: a population based survey in England
Source: BMC Cancer. 2019 Jun 11;19:566. doi: 10.1186/s12885-019-5787-x (PMC6561035; doi:10.1186/s12885-019-5787-x)
Supplement: Supplementary file 1 — Table S1. Unadjusted and adjusted odds ratios (OR), 95% confidence intervals (95% CI), and significance values for having never been screened (vs. having been screened at least once) by total CASS (cancer stigma) score for cervical (N = 681), breast (N = 326) and colorectal cancer screening (N = 371). (DOCX 14 kb) [file 12885_2019_5787_MOESM1_ESM.docx]

# Additional file 1

“Cancer stigma and cancer screening attendance: a population based survey in England” by Charlotte Vrinten, Ailish Gallagher, Jo Waller, and Laura AV Marlow.

Table S1. Unadjusted and adjusted odds ratios (OR), 95% confidence intervals (95% CI), and significance values for having never been screened (vs. having been screened at least once) by total CASS (cancer stigma) score for cervical (N=681), breast (N=326) and colorectal cancer screening (N=371).

|  | Unadjusted | | Adjusted | |
| --- | --- | --- | --- | --- |
|  | OR (95% CI) | p-value | OR (95% CI) | p-value |
| Cervical screening | 2.24 (1.54-3.26) | P<.001 | 2.02 (1.37-2.98)* | P<.001 |
| Breast screening | 2.44 (1.29-4.61) | P=.006 | 2.02 (1.05-3.88)* | P=.036 |
| Bowel screening | 1.71 (1.14-2.58) | P=.010 | 1.72 (1.13-2.63)** | P=.012 |

*Adjusted for age, ethnicity and social grade

**Adjusted for age, gender, ethnicity, and social grade.
